# Gut microbiota is associated with persistence of longer-term BNT162b2 vaccine immunogenicity

**DOI:** 10.3389/fimmu.2025.1534787

**Published:** 2025-02-27

**Authors:** Ho Yu Ng, Yunshi Liao, Ching Lung Cheung, Ruiqi Zhang, Kwok Hung Chan, Wai-Kay Seto, Wai K. Leung, Ivan F. N. Hung, Tommy T. Y. Lam, Ka Shing Cheung

**Affiliations:** ^1^ Li Ka Shing Faculty of Medicine, The University of Hong Kong, Hong Kong, Hong Kong SAR, China; ^2^ State Key Laboratory of Emerging Infectious Diseases, School of Public Health, The University of Hong Kong, Hong Kong, Hong Kong SAR, China; ^3^ Department of Pharmacology and Pharmacy, School of Clinical Medicine, The University of Hong Kong, Queen Mary Hospital, Hong Kong, Hong Kong SAR, China; ^4^ Laboratory of Data Discovery for Health Limited, 19W Hong Kong Science & Technology Parks, Hong Kong, Hong Kong SAR, China; ^5^ Department of Medicine, School of Clinical Medicine, The University of Hong Kong, Queen Mary Hospital, Hong Kong, Hong Kong SAR, China; ^6^ Department of Microbiology, School of Clinical Medicine, The University of Hong Kong, Queen Mary Hospital, Hong Kong, Hong Kong SAR, China; ^7^ Centre for Immunology & Infection Limited, 17W Hong Kong Science & Technology Parks, Hong Kong, Hong Kong SAR, China

**Keywords:** gut microbiota, vaccine, COVID-19 vaccine, vaccine immunogenicity, BNT162b2 (Pfizer-BioNTech)

## Abstract

**Introduction:**

BNT162b2 immunogenicity wanes with time and we investigated association between gut microbiota and longer-term immunogenicity.

**Methods:**

This cohort study prospectively recruited adult BNT162b2 two-dose recipients from three vaccination centers in Hong Kong. Blood samples were collected at baseline and day 180 after first dose, and tested for neutralizing antibodies (NAb) against receptor-binding domain (RBD) of wild type SARS-CoV-2 virus using chemiluminescence immunoassay. Shotgun DNA metagenomic sequencing was performed to characterize baseline stool microbiome. Baseline metabolites were measured by gas and liquid chromatography-tandem mass spectrometry (GC-MS/MS and LC-MS/MS). Primary outcome was persistent high NAb response (defined as top 25% of NAb level) at day 180. Putative bacterial species and metabolic pathways were identified using linear discriminant analysis [LDA] effect size analysis. Multivariable logistic regression adjusting for clinical factors was used to derive adjusted odds ratio (aOR) of outcome with bacterial species and metabolites.

**Results:**

Of 242 subjects (median age: 50.2 years [IQR:42.5-55.6]; male:85 [35.1%]), 61 (25.2%) were high-responders while 33 (13.6%) were extreme-high responders (defined as NAb≥200AU/mL). None had COVID-19 at end of study. *Ruminococcus bicirculans* (log_10_LDA score=3.65), *Parasutterella excrementihominis* (score=2.82) and *Streptococcus salivarius* (score=2.31) were enriched in high-responders, while *Bacteroides thetaiotaomicron* was enriched in low-responders (score=-3.70). On multivariable analysis, bacterial species (*R. bicirculans*–aOR: 1.87, 95% CI: 1.02-3.51; *P. excrementihominis*–aOR: 2.2, 95% CI: 1.18-4.18; S. *salivarius*–aOR: 2.09, 95% CI: 1.13-3.94) but not clinical factors associated with high response. *R. bicirculans* positively correlated with most metabolic pathways enriched in high-responders, including superpathway of L-cysteine biosynthesis (score=2.25) and L-isoleucine biosynthesis I pathway (score=2.16) known to benefit immune system. Baseline serum butyrate (aOR:10.00, 95% CI:1.81-107.2) and isoleucine (aOR:1.17, 95% CI:1.04-1.35) significantly associated with extreme-high vaccine response.

**Conclusion:**

Certain gut bacterial species, metabolic pathways and metabolites associate with longer-term COVID-19 vaccine immunogenicity.

## Introduction

Studies showed that there was a steady decline of antibody levels among COVID-19 vaccine recipients (1, 2), and vaccine effectiveness against infection decreased significantly from 92% at 15-30 days to 47% at 121-180 days (3). The durability of vaccine immunogenicity is in large contributed by the persistence of antibody levels, which in turn is affected by the amount of long-lived plasma cells that secrete the antibodies, and also by memory B cells which are responsible for secondary immune response during re-exposure to the pathogen (4, 5). Nevertheless, factors influencing the durability of vaccine immunogenicity are currently underinvestigated. While certain factors, such as age, comorbidities (such as obesity and diabetes mellitus) and prior infection were more established in affecting vaccine durability (6), the role of the gut microbiome in vaccine durability is relatively less understood and explored.

Gut microbiota modulates immune response toward various vaccines including influenza and oral rotavirus (7), possibly by secreting immunostimulatory short chain fatty acids (SCFAs) (8), secondary bile acids (9), lipopolysaccharides (LPS) (10), flagellin (11), and peptidoglycans (12). Notably, a randomized controlled trial (RCT) showed that antibiotic-induced gut microbiota dysbiosis attenuated the level of influenza vaccine-induced antibodies, increased inflammatory signaling and disturbed plasma metabolome (9). Moreover, it has been suggested that gut microbiota was linked to memory B cells differentiation, possibly through influencing the formation of germinal centers in the gut which was important for B cell differentiation into memory B cells (13).

Emerging evidence has reported the potential role of gut microbiota in COVID-19 vaccine immunogenicity (**Supplementary Table 1**) (14). For instance, two studies of mostly non-immunocompromised subjects reported that enrichment of certain bacterial species associated with higher antibody level, such as *Eubacterium rectale* and *Roseburia faecis* in one (15), and *Collinsella aerofaciens, Fusicatenibacter saccharivorans* in another (16). In another study conducted in infliximab-treated inflammatory bowel disease (IBD) patients, *Bilophila* was associated with above average immune response after second dose of vaccination with BNT162b2 or ChAdOx1 (17). However, these studies measured antibody response within a relatively short time frame from vaccination (15–18). Moreover, some had small sample sizes (17–19) and might even be focused on immunocompromised patient groups that did not reflect the healthy general population (17, 18). Some employed 16S rRNA sequencing which had lower resolution than shotgun metagenomic sequencing (17–19). Factors that might affect the microbiota, such as diet, were also often not adjusted (15–20). Therefore, the potential role of gut microbiota in persistence of longer-term immunogenicity toward COVID-19 vaccine in healthy adult subjects deserves further investigation.

We conducted this prospective cohort study to investigate association between gut microbiota composition and BNT162b2 immunogenicity in immunocompetent adults at 6 months after vaccination.

## Methods

### Study design and participants

This was a prospective cohort study recruiting adult subjects receiving two doses of BNT162b2 vaccines containing mRNA encoding viral spike (S) protein of SARS-CoV-2 from three vaccination centers in Hong Kong (Sun Yat Sen Memorial Park Sports Centre, Ap Lei Chau HKU Vaccination Centre and Queen Mary Hospital) between May and August 2021. Exclusion criteria included age <18 years, IBD, immunocompromised status including post-transplantation and immunosuppressives/chemotherapy, other medical diseases (cancer, hematological, rheumatological and autoimmune diseases) and those with prior COVID-19 identified from history or the presence of antibodies to SARS-CoV-2 nucleocapsid (N) protein. This study was approved by the Institutional Review Board (IRB) of the University of Hong Kong and Hospital Authority Hong Kong West Cluster (UW 21-216). All participants provided written informed consent for participation in this study.

### Collection of demographics, blood and stool samples

Basic demographics including age, sex, lifestyle factors (level of exercises, diet and smoking), overweight or obese (OWOB) (21–23), diabetes mellitus (DM) (21), hepatic steatosis (24, 25) as well as any use of antibiotics (26) and proton pump inhibitors (PPIs) within six months before vaccination were collected. Adequate level of exercises is defined as meeting WHO recommendation of at least 150 to 300 minutes of moderate-intensity aerobic physical activity per week, or at least 75 to 150 minutes of vigorous-intensity physical activity per week (27). Diet quality is assessed using the rapid Prime Diet Quality Score (rPDQS), which is a validated diet quality screener with a score ranging from 0 to 52, and a higher score reflecting better diet quality (28). We created tertile groups, and compared the highest tertile with the lowest/middle tertile similar to other studies (29).

Subjects received two doses of intramuscular BNT162b2 (0.3mL) 3 weeks apart as recommended by local health authority. Blood samples were collected (i) before vaccination (baseline) and (ii) at 180days after first dose. Baseline stool samples were collected in OMNIgene tube before vaccine administration and stored at -80°C until total genomic DNA was extracted from them using Qiagen QIAamp DNA stool Mini Kit (Qiagen, Hilden, Germany) according to the manufacturer’s instructions. Genomic DNA was then subjected to library preparation for shotgun metagenomic sequencing using Nextera DNA Library Prep Kit (Illumina, California, USA). In brief, genomic DNA was first fragmented and tagged with adapter sequences by engineered transposome. Index adapter sequences were then added to these tagged DNA using limited cycle PCR. After amplification, PCR amplicons were purified using AMPure XP beads (Beckman-Coulter). Quality of the DNA library was first assessed by a Qubit fluorometer (Thermo Fisher Scientific), then by a Bioanalyzer (Agilent Technologies). After library preparation, next-generation shotgun metagenomic sequencing was performed by the Illumina NovaSeq 6000 platform (Illumina, San Diego, US) running at paired-end 150 bp, resulting in 10 Gb raw data per sample.

We employed a cost-effective extreme case-control design in selecting the extreme high responders (defined as NAb > 200 AU/mL) and extreme low responders (defined as NAb < 50 AU/mL) (30), and performed targeted metabolomics on selected metabolites, which were SCFAs (acetate, propionate and butyrate) due to more evidence in association with vaccine immunogenicity from literature, as well as L-isoleucine which was implicated in our metabolic pathway analysis. Extreme case-control design utilizes an extreme-value sampling design approach to select individuals with extremely large or small values of the primary outcome for exposure data collection (30). It has been shown that such an approach with appropriate statistical analysis could control type I error well and achieve cost-effectiveness (30, 31). Furthermore, the main purpose of this design was to exclude participants with highly similar NAb levels in the intermediate range which would introduce noise into our analysis. Therefore, we selected an arbitrary cut-off of NAb ≥200 AU/mL and NAb<50AU/mL to only include participants who were in the extreme-high range and extreme-low range into analysis to further enhance the associations detected (32). Baseline serum SCFAs (acetate, propionate and butyrate) as well as L-isoleucine were measured using gas chromatography-tandem mass spectrometry (GC-MS/MS) and liquid chromatography-tandem mass spectrometry (LC-MS/MS), respectively. Detailed steps of GC-MS/MS and LC-MS/MS can be found in **Supplementary File**.

Vaccine immunogenicity was determined in terms of neutralizing antibody (NAbs) against SARS-CoV-2 receptor-binding domain (RBD). NAb level is a surrogate marker of vaccine effectiveness (33) that predicts protection from symptomatic COVID-19 (34, 35). Although the gold standard to measure NAb level is live virus microneutralization assay (vMN), this test must be conducted under biosafety level-3 containment and therefore is not widely applicable to daily clinical practice, in particular during period of infection outbreak. Our previous study showed that a surrogate NAb assay, performed using the new version of the iFlash-2019-nCoV NAb kit (chemiluminescent microparticle immunoassay; Shenzhen YHLO Biotech Co, Ltd., Shenzhen, China), had good diagnostic performance (sensitivity: 98%, specificity: 95%, positive predictive value: 98% and negative predictive value: 94%) and agreement of 94% relative to vMN assay (36).

In the current study, testing for NAb was performed using iFlash-2019-nCoV NAb kit. Briefly, serum samples and a reagent pack with 2019-nCoV RBD antigen (30KD)-coated paramagnetic microparticles and acridinium ester-labeled ACE2 conjugate were placed in sample loading area and reagent loading area respectively (36). The iFlash system then automatically performed all functions and measured signals elicited from chemiluminescent reactions. NAb seropositivity was defined as ≥15 AU/mL.

### Primary outcome of interest

Primary outcome of interest was persistent high NAb response at day 180. We defined top 25% of NAb (i.e. above 75 percentile) as high NAb response similar to the study by Tang et al. (16) NAb seropositivity was not chosen as primary outcome because 95.9% of the cohort remained seropositive at day 180.

### Bioinformatics analysis

Raw NGS reads were processed by fastp v0.20.1 (37) to quality and adapter trimming to remove sequencing adapters and bases with poor quality. Trimmed reads were subjected to host sequence removal by Bowtie2 (38) to map reads against human reference genome GRCh38.p13. Composition of microbial communities at species level and functional profile in each sample were inferred from the cleaned reads using MetaPhlAn (v3.0) (39) and HUMAnN (v3.0) (40) respectively. Estimation of species coverage and relative abundance was determined. Low-abundance taxa were not excluded from analysis as it has been shown that low-abundance bacteria could contribute substantially to host phenotypes (41) and there is no consensus in the analytical approach as to which levels filtering methods should be applied to remove low-abundance taxa, which would influence the results of downstream analyses (42). Instead, we incorporated negative control sample into sequencing to make sure the inferred microbiome taxa were not derived from contaminants introduced during sampling, lab work, or sequencing.

### Statistical analysis

All statistical analyses were conducted with R version 4.2.2 (R Foundation for Statistical Computing, Vienna, Austria) statistical software. Data was displayed as median (interquartile range [IQR]) for continuous variables, and as number of patients (percentage) for categorical variables. The Mann-Whitney U test and the Chi-square test or Fisher exact test was used for two continuous variables and categorical variables respectively.

Alpha-diversity in terms of observed species, Shannon and Simpson index was computed using *vegan* package in R Studio, and compared using Wilcoxon signed-rank test. Beta-diversity including Bray-Curtis compositional dissimilarity was compared using non-metric multidimensional scaling (NMDS). Permutational multivariate analysis of variance (PERMANOVA) was used to compare microbial communities of different samples. Putative gut bacterial species and metabolic pathways with an absolute value of linear discriminant analysis (LDA) score ≥ 2 were identified using LEfSe (linear discriminant analysis effect size). The median was used to define a high relative abundance of a particular bacterial species.

A univariate logistic and linear regression model was used to estimate odds ratio (OR) and beta coefficients, respectively, of high-response with the various aforementioned clinical factors and with a high relative abundance of putative gut bacterial species. A multivariable logistic regression model was used to estimate adjusted OR (aOR) of high response with clinical factors and putative bacterial species of p<0.15 on univariate logistic regression analysis as in previous study (43). The performance of the variables with p<0.15 in either univariate logistic regression or univariate linear regression in predicting vaccine response was assessed by random forest machine learning model to derive the area under receiver operating characteristic curve (AUC). Details of the machine learning model can be found in **Supplementary Methods**.

To explore the relationship between DM and *P. excrementihominis* and vaccine immunogenicity, we stratified the subjects based on the baseline relative abundance of *P. excrementihominis* and DM status, and assigned four groups (High abundance-Non-DM, High abundance-DM, Low abundance-Non-DM, Low abundance-DM) to perform trend test (p_trend_). Similarly, to explore the relationship between OWOB and *P. excrementihominis* and vaccine immunogenicity, we stratified the subjects based on the baseline relative abundance of *P. excrementihominis* and OWOB status, and assigned four groups (High abundance-Normal weight, High abundance-OWOB, Low abundance-Normal weight, Low abundance-OWOB) to perform trend test (p_trend_).

Spearman’s correlation tests were used to analyze correlation among continuous variables. False discovery rate (FDR) was used to correct for multiple comparisons in multiple hypothesis testing, including during LefSe analysis (44).

For the analysis of metabolites, multivariable logistic regression was used to evaluate the association of metabolites with extreme high response. To achieve an alpha value of 0.05 and a power of at least 80% to detect an association if the metabolite has an odds ratio of at least 1.5, the sample size will be 70 for the extreme case-control design.

A two-sided p-value ≤0.05 was considered as statistically significant, while a p-value ≤ 0.1 was considered borderline significant.

## Results

### Baseline characteristics

We recruited 242 eligible adults who had received two doses of BNT162b2. The median age was 50.2 years (IQR:42.5-55.6; range 18-75), and 85 (35.1%) were male. 232 (95.9%) remained seropositive (≥15 AU/mL) at day 180 (median NAb level:55.2 AU/mL; IQR:30.8-123.6). 61 (25.2%) were classified as persistent high-responders (median NAb level:205.4 AU/mL; IQR:164.6-291.3) while 181 (74.8%) were low-responders (median NAb level:40.9 AU/mL; IQR:25.8-66.4). There were 119 (51.1%) subjects who had adequate level of exercises as per WHO recommendations, and 97 (41.6%) had rPDQS at top tertile. Additionally, there were 134 (55.4%) subjects who were OWOB, and 16 (6.6%) had DM. Baseline characteristics were comparable between the high- and low-responders (all p>0.05) (**Table 1**). At day 180, none had SARS-CoV-2 after they received two doses of BNT162b2.

**Table 1:** Baseline characteristics between persistent high- and low-responders of BNT162b2.

Characteristics	Whole cohort (N = 242)	Low-responder (N = 181)	High-responder (N = 61)	p-value
Age ≥ 55 years (n, %)	67 (27.7%)	55 (30.4%)	12 (19.7%)	0.106
Male sex (n, %)	85 (35.1%)	63 (34.8%)	22 (36.1%)	0.859
Adequate level of exercises (n, %)^†, #^	119 (51.1%)	85 (49.1%)	34 (56.7%)	0.314
Diet (rPDQS score ≥ 3^rd^ tertile) (n, %)^#^	97 (41.6%)	74 (42.8%)	23 (38.3%)	0.548
Smoking (n, %)	12 (5.0%)	11 (6.1%)	1 (1.6%)	0.304
Overweight or obese (n, %)	134 (55.4%)	101 (55.8%)	33 (54.1%)	0.817
Diabetes mellitus (n, %)	16 (6.6%)	15 (8.3%)	1 (1.6%)	0.079
Triglycerides (mmol/L)	0.9 (0.7, 1.3)	0.9 (0.7, 1.2)	0.9 (0.7, 1.4)	0.814
Total cholesterol (mmol/L)	4.8 (4.2, 5.5)	4.8 (4.3, 5.4)	4.8 (4.0, 5.7)	0.804
LDL (mmol/L)	2.8 (2.3, 3.2)	2.8 (2.3, 3.2)	2.7 (2.3, 3.4)	0.850
Non-alcoholic fatty liver disease (n, %)	91 (37.6%)	67 (37.0%)	24 (39.3%)	0.745
Proton pump inhibitors use* (n, %)	26 (10.7%)	21 (11.6%)	5 (8.2%)	0.458
Antibiotic use* (n, %)	21 (8.7%)	18 (9.9%)	3 (4.9%)	0.228

High neutralizing antibody response was defined as the top 25% (i.e. above 75 percentile).

*usage for any duration within six months before vaccination.

^†^adequate level of exercises is defined as meeting WHO recommendation (at least 150-300 minutes of moderate-intensity, or 75-150 minutes of vigorous-intensity aerobic exercise per week).

^#^Total 9 missing data: 8 in low-responder group, 1 in high-responder group.

LDL, low-density lipoprotein; WHO, World Health Organization; rPDQS, rapid prime diet quality score.

### Clinical factors associated with persistent high response

On univariate logistic regression, age ≥ 55 years old and DM were negatively associated with persistent high vaccine response with borderline statistical significance (**Table 2**). On univariate linear regression, OWOB and DM were negatively associated with persistent high vaccine response with borderline statistical significance (**Supplementary Table 2**). Other factors including lipid profile, lifestyle factors (level of exercises, diet and smoking), and antibiotic use were not significantly associated with persistent high vaccine response on both univariate logistic and univariate linear regression (**Table 2**, **Supplementary Table 2**).

**Table 2:** Univariate and multivariate logistic regression between high-vaccine response and a combination of clinical factors and bacterial species.

	Univariate logistic regression			Multivariate logistic regression		
	OR	95% CI	p-value	aOR	95% CI	p-value
Clinical factors						
Age ≥ 55 years	0.56	0.27, 1.11	0.109	0.63	0.29, 1.30	0.224
Male sex	1.06	0.57, 1.92	0.859			
Adequate level of exercises*	1.35	0.75, 2.46	0.315			
Diet (rPDQS score at 3^rd^ tertile)	0.83	0.45, 1.51	0.548			
Smoking	0.26	0.01, 1.37	0.198			
Overweight or obese	0.93	0.52, 1.68	0.817			
Diabetes mellitus	0.18	0.01, 0.94	0.105	0.20	0.01, 1.08	0.132
Triglycerides	1.32	0.81, 2.13	0.250			
Total cholesterol	1.05	0.77, 1.42	0.778			
Low-density lipoprotein	1.13	0.78, 1.62	0.506			
Hepatic steatosis	1.10	0.60, 1.99	0.746			
Proton pump inhibitor use	0.68	0.22, 1.76	0.460			
Antibiotic use	0.47	0.11, 1.45	0.238			
High relative abundance of gut bacterial species^†^						
*Ruminococcus bicirculans*	1.95	1.08, 3.57	0.028	1.87	1.02, 3.51	0.046
*Parasutterella excrementihominis*	2.14	1.19, 3.94	0.013	2.20	1.18, 4.18	0.014
*Streptococcus salivarius*	1.78	0.99, 3.25	0.056	2.09	1.13, 3.94	0.020
*Bacteroides thetaiotaomicron*	0.67	0.37, 1.2	0.184			

*Adequate level of exercises is defined as meeting WHO recommendation (at least 150-300 minutes of moderate-intensity, or 75-150 minutes of vigorous-intensity aerobic exercise per week).

^†^High relative abundance is defined as relative abundance higher than median of the cohort.

95% CI, 95% confidence interval; OWOB, overweight or obese; WHO, World Health Organization; rPDQS, rapid prime diet quality score; NAb, neutralizing antibodie.

### Baseline gut microbiota composition was associated with persistent high response to BNT162b2 at day 180

There was no significant difference in alpha diversity (richness, Shannon and Simpson all p>0.05; **Supplementary Figure 1**) and beta diversity (PERMANOVA analysis, p=0.270; **Supplementary Figure 2**) between high- and low-responders.

**Figure 1:**
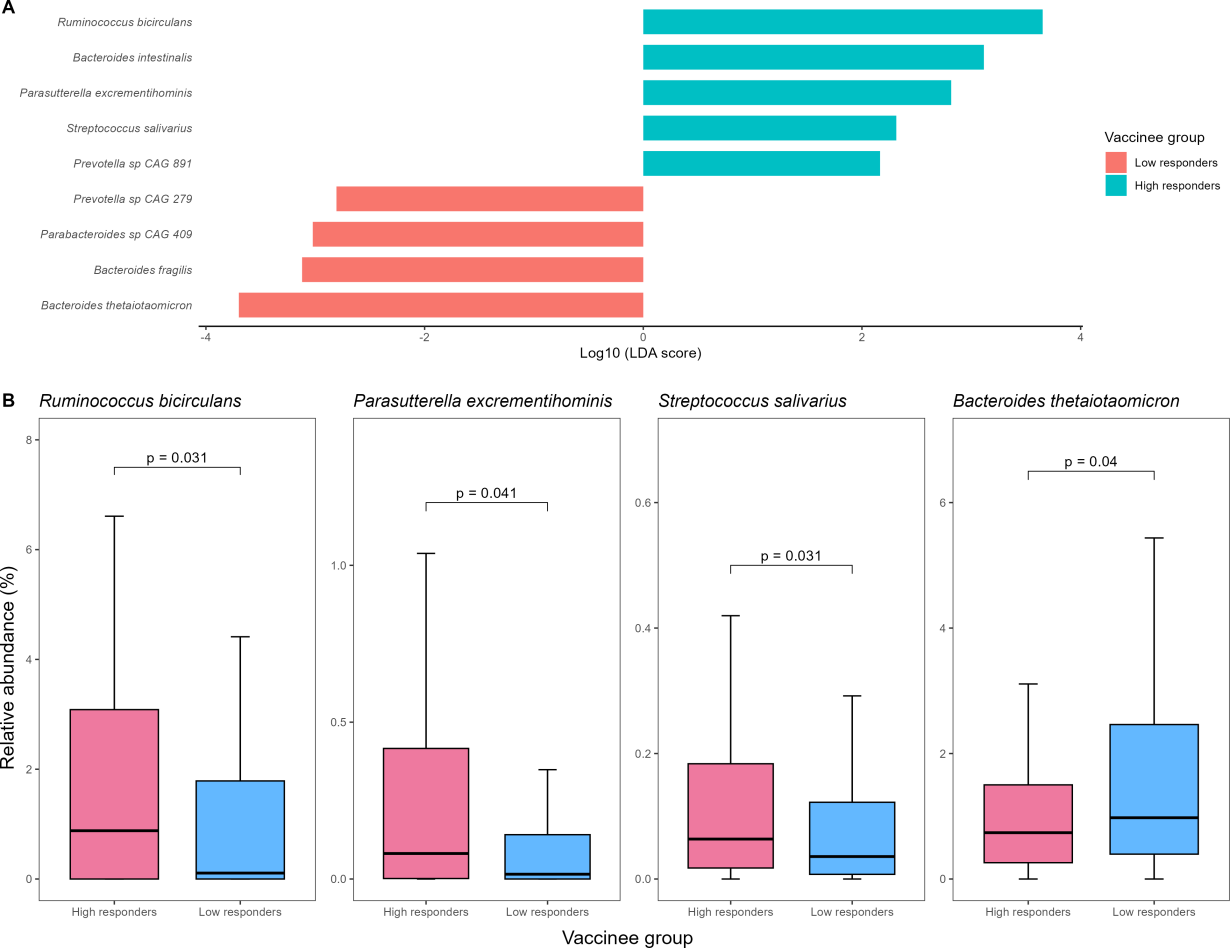
**(A) ** Baseline gut bacterial species enriched in high- vs low-vaccine responders detected by LEfSe **(B)** Comparison of relative abundances of putative baseline gut bacterial species identified on LEfSe analysis between high- and low-vaccine responders. LEfSe, linear discriminant analysis effect size.

Using LEfSe analysis, we found that five bacterial species (namely *Ruminococcus bicirculans, Bacteroides intestinalis, Parasutterella excrementihominis, Streptococcus salivarius*, and *Prevotella* sp *CAG 891*) and four (namely *Prevotella* sp *CAG 279, Parabacteroides* sp *CAG 409, Bacteroides fragilis*, and *Bacteroides thetaiotaomicron*) were enriched in high- and low-responders in baseline gut microbiome respectively (**Figure 1**). Among them, *R. bicirculans, P. excrementihominis, S. salivarius* and *B. thetaiotaomicron* were not zero-inflated (i.e. their median relative abundance was not equal to zero). Specifically, *R. bicirculans* (log_10_LDA score=3.65; 0.9% vs 0.1%; p=0.031)*, P. excrementihominis* (log_10_LDA score=2.82; 0.08% vs 0.02%; p=0.040) and *S. salivarius* (log_10_LDA score=2.31; 0.06% vs 0.04%; p=0.031) were enriched in persistent high-responders, while *B. thetaiotaomicron* was enriched in low-responders (log_10_LDA score=-3.70; 2.1% vs 1.1%; p=0.040) (**Figures 1A, B**).

On univariate logistic regression, a higher abundance of *R. bicirculans* (OR:1.95; 95% CI:1.08-3.57), *P. excrementihominis* (OR:2.14; 95% CI:1.19-3.94) and *S. salivarius* (OR:1.78; 95% CI:0.99-3.25) was associated with high-response (all p<0.10) (**Table 2**).

We then incorporated clinical (age ≥ 55 years and DM) and the three bacterial species factors with p<0.15 on univariate logistic regression into multivariate logistic regression. Only bacterial species (*R. bicirculans–*aOR:1.87, 95% CI:1.02-3.51; *P. excrementihominis–*aOR:2.2, 95% CI:1.18-4.18; *S. salivarius–*aOR:2.09, 95% CI:1.13-3.94) but not clinical factors (age ≥55 years*–*aOR:0.63, 95% CI:0.29-1.30; DM*–*0.20, 95% CI:0.01-1.08) were associated with persistent high-response (**Table 2**). Sensitivity analysis incorporating OWOB also yielded similar results (**Supplementary Table 3**). The clinical factors (age ≥ 55 years, DM and OWOB) and the and the three bacterial species factors with p<0.15 on either univariate logistic regression or univariate linear regression collectively predict vaccine response with AUC of 0.712 by random forest machine learning model (**Supplementary Figure 3**).

As DM and OWOB were potential factors affecting vaccine immunogenicity on univariate analysis, we further investigated the relative abundances of bacterial markers in patient cohorts, as well as the role of these metabolic factors as an effect modifier of bacteria-immune response relationship. **Supplementary Figure 4** shows that there was no significant difference in relative abundances of bacterial markers between non-DM and DM subjects as well as non-OWOB subjects and OWOB subjects, except for *R. bicirculans* which was significantly more abundant in non-DM subjects than DM subjects (0.27% vs 0%;p=0.044). Non-DM subjects with a high relative abundance of *P. excrementihominis* had significantly higher median NAb level than non-DM subjects with low abundance of *P. excrementihominis* (81.7 [IQR:37.8-165.0] vs 45.1 AU/mL [IQR:27.6-91.6];p=0.003) (**Supplementary Figure 5A**). When stratified by baseline relative abundance of *P. excrementihominis* and DM status, and taking high abundance-non DM as reference, there was a decreasing trend of NAb level at day 180 (p-trend=0.022, **Supplementary Table 4**). Similarly, non-OWOB subjects with high relative abundance of this species compared with non-OWOB subjects with low abundance of this species (101.0 [IQR:46.5-165.0] vs 42.7 AU/mL [IQR:26.8-91.6];p=0.009) (**Supplementary Figure 6A**). When stratified by baseline relative abundance of *P. excrementihominis* and OWOB status, and taking high abundance-normal weight as reference, there was a significant decreasing trend of NAb level at day 180 (p-trend=0.009, **Supplementary Table 5**).

We then considered having high relative abundance of *P. excrementihominis*, non-OWOB and non-DM as favorable factors, and found that subjects who had all three favorable factors had a significantly higher median NAb level (106.0 AU/mL [IQR:48.3-167.0]) than those with just two (55.1 [IQR:28.9-122.0];p=0.015), one (46.0 AU/mL [IQR:28.6-81.9];p=0.007) or no protective factors (20.6 AU/mL [IQR:19.7-35.0];p=0.014) (**Supplementary Figure 7**).

On the other hand, there were no similar observations among other three putative bacterial markers with either DM or OWOB on NAb level (**Supplementary Figures 5B–D** and **Supplementary Figures 6B–D**). While non-DM subjects with high relative abundance of *S. salivarius* had significantly higher median NAb level than DM subjects with low relative abundance of this species (60.0 [IQR:30.8-160.0] vs 25.9 AU/mL [IQR:19.7-35.2];p=0.026), there was no similar observations in non-OWOB subjects with high relative abundance of this species compared with OWOB subjects with low relative abundance of this species.

Additionally, we conducted subgroup analysis based on sex, and found that in male subjects, high abundance of *P. excrementihominis* were associated with high vaccine response at day 180 (aOR=4.59, 95% CI: 1.51-15.70, p=0.010) (**Supplementary Table 6**). On the other hand, in female subjects, high abundance of *R. bicirculans* (aOR=2.36, 95% CI: 1.09-5.35, p=0.034) and *S. salivarius* (aOR=2.29, 95% CI: 1.07-5.03, p=0.035) were associated with high vaccine response at day 180 (**Supplementary Table 6**).

### Association between metabolic pathways and persistent vaccine immunogenicity at day 180

We then investigated the metabolic pathways in baseline gut microbiome and found that six and five pathways were enriched in high- and low-responders respectively (**Supplementary Figure 8**). They were classified into “Biosynthesis” and “Degradation/Utilization/Assimilation” categories according to MetaCyc database (**Supplementary Table 7**). In persistent high-responders, enriched pathways included those related to energy production as well as amino acid biosynthesis, such as superpathway of L-cysteine biosynthesis (mammalian) (log_10_LDA score=2.25;p=0.005) and superpathway of L-isoleucine biosynthesis I (log_10_LDA score=2.16;p=0.020).

### Correlation between gut microbiota and metabolic pathways on vaccine immunogenicity

**Figure 2:**
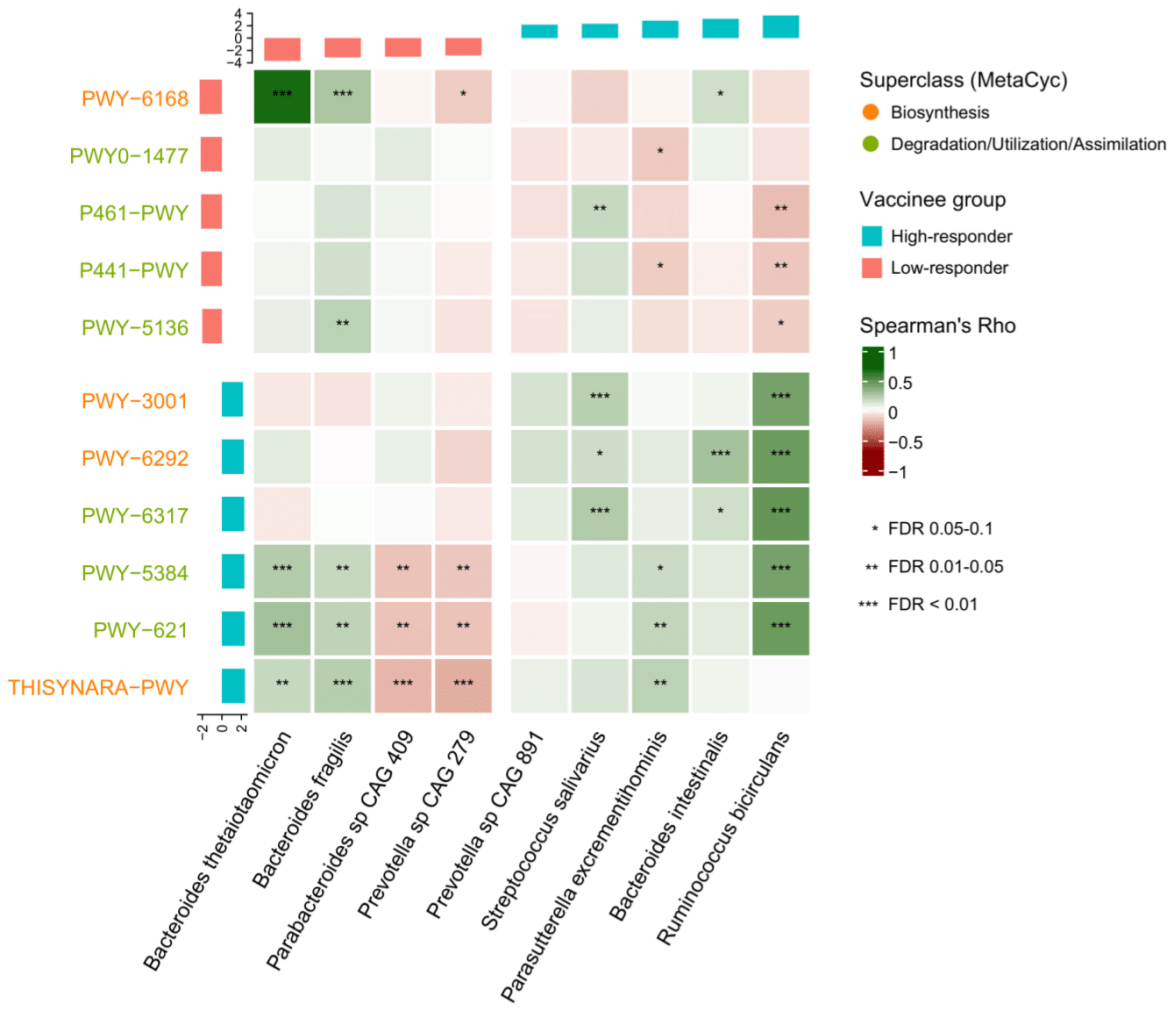
Spearman correlation between baseline metabolic pathways and gut bacterial species.

We performed Spearman’s correlation analysis between baseline gut bacterial species and metabolic pathways (**Figure 2** **).** Most of the metabolic pathways enriched in high-responders were positively associated with *R. bicirculans*. Notably, superpathway of L-cysteine biosynthesis (mammalian) and superpathway of L-isoleucine biosynthesis I showed positive correlation with *R. bicirculans* (Spearman’s r=0.45;p<0.001 and r=0.41;p<0.001 respectively) and *S. salivarius* (r=0.15;p=0.069 and r=0.21;p=0.007 respectively).

### Association between baseline serum metabolites and response to BNT162b2 at day 180

Thirty-three and 37 subjects were classified as extreme-high responders and extreme-low responders, respectively. Among them, 29 (41.4%) were male, and the median age was 50.2 years old (IQR:40.1-53.8). For SCFAs, butyrate was significantly associated with extreme high vaccine response (aOR:10.00, 95% CI:1.81-107.2; p=0.025), while propionate was of borderline significance (aOR:1.33, 95% CI: 0.97-1.92; p=0.091) and acetate did not show significant association (aOR:1.01, 95% CI:0.99-1.02; p=0.368). Baseline serum isoleucine was also significantly associated with extreme high vaccine response (aOR:1.17, 95% CI:1.04-1.35; p=0.016).

## Discussion

To our knowledge, our study was the first to show the association of baseline gut microbiota composition with persistently high immunogenicity toward BNT162b2 at 180 days post-vaccination. We identified four potential baseline microbial markers, namely *R. bicirculans, P. excrementihominis, S. salivarius* and *B. thetaiotaomicron*, as well as metabolic pathway markers that might predict longer-term vaccine immunogenicity. In particular, *R. bicirculans* positively correlated with most of the metabolic pathways enriched in persistent high-responders, highlighting it as a potential key bacterial species that might be beneficial to the immune system.

Gut microbiota is involved in modulating immune response toward vaccination via production of immunomodulatory metabolites. Some of these metabolites served as vaccine adjuvants through activating PRRs such as toll-like receptors (TLRs) or NOD-like receptors. TLR5-mediated sensing of flagellin enhances the presence of short-lived plasma cells and immune response to influenza vaccine (11). TLR-4 mediated sensing of bacterial LPS promotes type 1 T helper cells (Th1) and antibody production (10), while NOD2-mediated recognition of bacterial peptidoglycan contributes to the mucosal adjuvant activity of cholera toxin (12). Other key metabolites include short-chain fatty acids (SCFAs) and secondary bile acids. The former increases antibody production through promoting energy production in B cells (8), while the latter was negatively correlated with inflammatory signatures following influenza vaccination (9).

Additionally, the gut microbiota was implicated in promoting memory B cell formation, which is important for antibody durability. In mouse models, correction of gut microbiota dysbiosis were able to promote germinal center formation, which was required for memory B cell differentiation (45). Moreover, bacterial LPS could activate TLR4, which has been shown to enhance the persistence of germinal centers and early differentiation into long-lived memory cells in rhesus macaques (46). In humans, it has been shown that early colonization of microbiota such as *Bifidobacteria* in infants was associated with future increased frequency of CD27+ memory B cells (47, 48). Conversely, babies born to mothers with IBD were found to be depleted in *Bifidobacteria*, which was associated with fewer class-switched memory B cells in later timepoints (49). These suggest that the gut microbiota may have a role to play in promoting durable vaccine immunogenicity, which was largely contributed by memory B cell function.

**Figure 3:**
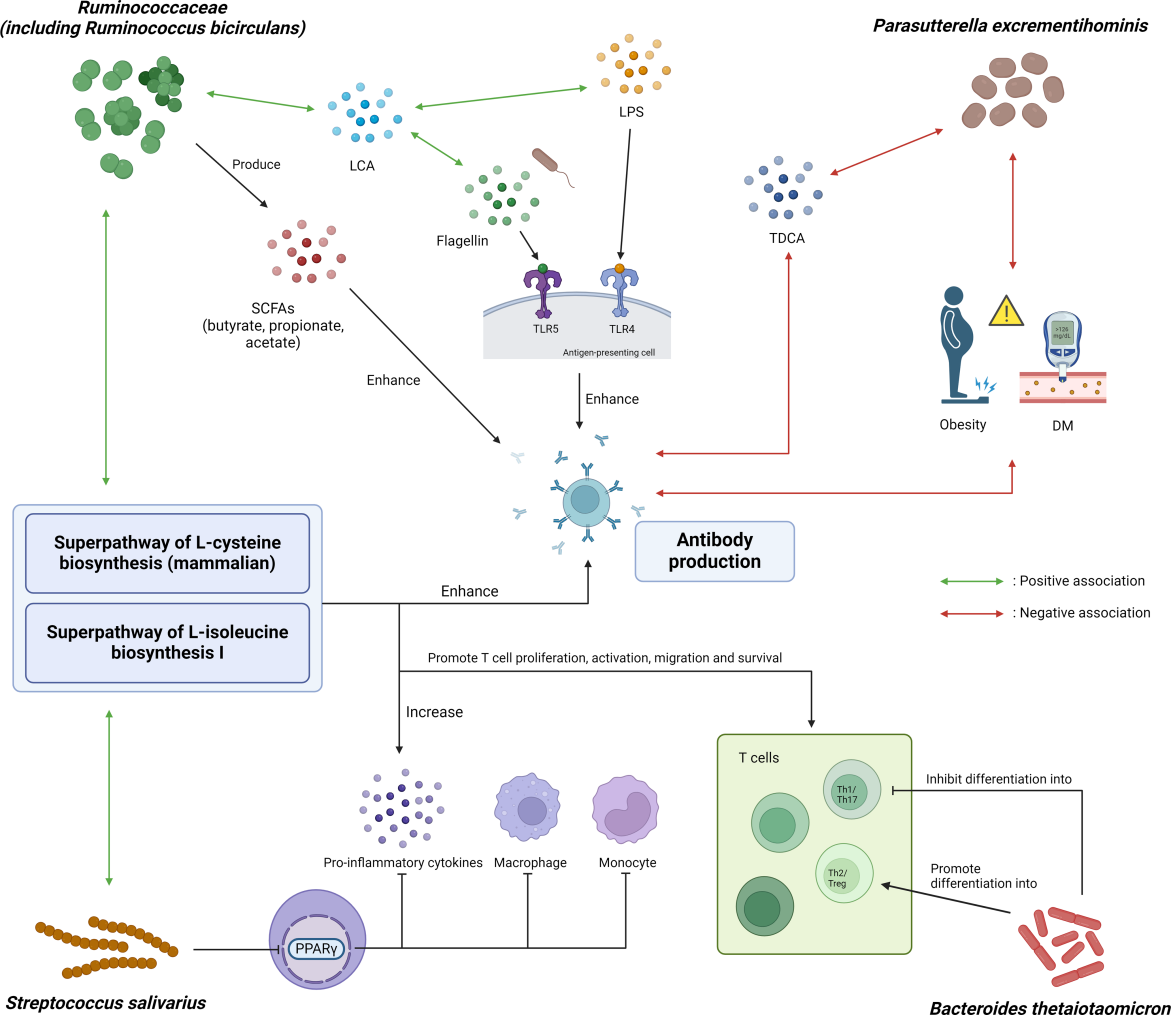
Graphical illustration of potential mechanistic links between microbial and metabolic markers with COVID-19 vaccine immunogenicity. *Ruminococcaceae*, including *Ruminococcus bicirculans*, can produce SCFAs such as butyrate, propionate and acetate, which in turn can enhance *in vivo* polyclonal antibody production. It is also positively associated with fecal lithocholic acid (LCA), which was shown to have positive association with flagellin and lipopolysaccharide (LPS) content in the stool after influenza vaccination. *Parasutterella excrementihominis* was negatively associated with obesity, diabetes mellitus and taurodeoxycholic acid (TDCA), all of which were associated with poorer response to vaccines. *Streptococcus salivarius* can inhibit monocytes, macrophages and production of pro-inflammatory cytokines through inhibiting PPARγ. *Bacteroides thetaiotaomicron* inhibits differentiation into Th1 cells which was important for protection against SARS-CoV-2, thereby attenuating antibody response to COVID-19 vaccine. Additionally, superpathway of L-cysteine biosynthesis (mammalian) and superpathway of L-isoleucine biosynthesis I were positively correlated with *R. bicirculans* and *S. salivarius*. Cysteine and isoleucine boost the immune system by promoting T cell proliferation, activation and survival, as well as enhancing the production of pro-inflammatory cytokines as well as antibody production. SCFA, short-chain fatty acid; LCA, lithocholic acid; LPS, lipopolysaccharide; TDCA, taurodeoxycholic acid; DM, diabetes mellitus; PPARγ, peroxisome proliferator-activated receptor gamma; TLR, toll-like receptor; Th1/2/17, T helper 1/2/17 cells; Treg, regulatory T cells.

On multivariable analysis, high abundance of *R. bicirculans, P. excrementihominis* and *S. salivarius* remained predictive of persistently high vaccine immunogenicity, while the clinical factors (age ≥ 55 years old and DM) were not. This suggests that high abundance of *R. bicirculans, P. excrementihominis* and *S. salivarius* in stool samples is a more important factor than routine clinical parameters in predicting vaccine immunogenicity. Consistently, the significant bacterial species identified from LEfSe analysis in our current study are capable of producing these immunomodulatory metabolites (**Figure 3**). Our metabolomics analysis also showed that baseline butyrate and propionate levels were associated with extreme high vaccine response at day 180 with statistical significance and borderline significance, respectively.

*Ruminococcacecae* produces SCFA including butyrate, propionate and acetate (50, 51). SCFAs can boost antibody production by stimulating expression of genes necessary for plasma cell differentiation, and increasing energy production through glycolysis, oxidative phosphorylation and fatty acid synthesis (8, 52). In particular, butyrate can also boost antibody production through upregulating follicular helper T cells which promote the activation and differentiation of plasma cells (52). Apart from producing acetate (53, 54), *R. bicirculans* is positively associated with the levels of fecal lithocholic acid (LCA) (55), which was positively associated with flagellin and LPS content in stool after influenza vaccination (9). P*. excrementihominis* was associated with a reduction in taurodeoxycholic acid (TDCA) in a mice study (56), which in turn was associated with poorer response to COVID-19 vaccination (including BNT162b2 vaccine) in infliximab-treated IBD patients (17). *S. salivarius* was shown to be able to inhibit peroxisome proliferator-activated receptor gamma (PPARγ) activation (57), which negatively regulates monocytes and macrophages and inhibits the production of pro-inflammatory cytokines (58).

On the other hand, *B. thetaiotaomicron*, which was enriched in low responders, may promote the preferential differentiation of anti-inflammatory Treg/Th2 cells while suppressing the relative differentiation of pro-inflammatory Th1/Th17 cells (59). Moreover, the polysaccharide A (PSA) of *B. thetaiotaomicron* promotes the function of Treg cells through interaction with toll-like receptor 2 (TLR2) (60). Th1 profile was favored following BNT162b2 vaccination and was important for protection against SARS-CoV-2 infection (61). Therefore, suppression of Th1 cells differentiation might attenuate the antibody response to BNT162b2. On the other hand, Treg cells suppresses B cell functions, including NAb production and immune memory (62). Conversely it has been shown in mouse models that depletion of Treg cells using anti-CD25 monoclonal antibody could induce more durable immunogenicity to BCG and hepatitis B vaccinations (63). Therefore, the preferential differentiation of Treg cells due to *B. thetaiotaomicron* might also have dampened the antibody response to BNT162b2.

Additionally, we found that superpathway of L-cysteine biosynthesis (mammalian) and superpathway of L-isoleucine biosynthesis I pathway were enriched in persistent high responders, and were positively correlated with *R. bicirculans* and *S. salivarius* (**Figure 3**). The metabolic activities of *Ruminococcus bicirculans* can produce H_2_S and acetyl-CoA (64), which provide materials and an energy source for L-cysteine biosynthesis through the superpathway of L-cysteine biosynthesis (fungi). Acetyl-CoA also contributes to the synthesis of L-isoleucine as a material, along with oxaloacetate, through the superpathway of L-isoleucine biosynthesis I. Extracellular cysteine was important for T cell proliferation, activation and survival (65). Cysteine transporters were strongly upregulated during T cell activation, and DNA synthesis in T cells was dependent on cysteine (66, 67). Extracellular cysteine could also affect the level of intracellular glutathione, as well as the activity of NFκB pathway which was involved in the secretion of inflammatory cytokines (68). Deficiency of extracellular cysteine was associated with immunodeficiency-related conditions, including acquired immune deficiency syndrome (AIDS) and a variety of cancers (65, 69). Isoleucine is a branched chain amino acid (BCAA) which was important for immune cell proliferation and function as well as production of pro-inflammatory cytokines (70, 71), and was greatly incorporated into lymphocytes (72). Isoleucine could induce the expression of β-defensins (70, 73), which was involved in activating IFN-γ (74). IFN-γ was in turn positively correlated with SARS-CoV-2 neutralizing antibody titers (75). In another study, isoleucine supplementation resulted in increased production of immunoglobulins, RV-specific antibodies and cytokines in the intestines and serum of RV-infected piglets (76), supporting its potential beneficial role in boosting immunity against viral infections. Our current study also showed that the baseline isoleucine was significantly associated with extreme high vaccine response at day 180.

We also investigated the interaction between significant bacterial species and metabolic factors with NAb level at six months. Interestingly, we found that different combinations of DM status, OWOB status and baseline abundance of *P. excrementihominis* were associated with different levels of vaccine response (**Figure 3**). Those subjects who were non-DM and non-OWOB with a higher relative abundance of *P. excrementihominis* had significantly higher NAb level than those with only one or even without these protective factors. Obesity (21–23) and DM (21) are associated with lower vaccine immunogenicity to COVID-19 vaccines (21). *Parasutterella* was shown to be negatively associated with obesity in both mice models (77) and in Chinese adults (78). *Parasutterella* was also found to be enriched in healthy pregnant women compared to those with gestational DM (79), and several RCTs found that these interventions were able to reverse gut dysbiosis in DM patients by increasing abundance of *Parasutterella (* 80–83). This might explain the interactions among these three protective factors, and the synergistic effect they exert on vaccine response.

It should be noted that other studies have identified different bacterial markers that might also predict COVID-vaccine immunogenicity. The discrepancy in results might be explained by inherent variations in gut microbiota composition across different populations due to factors including diet, lifestyle and socioeconomic status (7), and also by heterogenous study designs in terms of sample size and population, vaccine type, sequencing method, as well as the timepoint of measuring vaccine immunogenicity. Existing studies that investigated the association of gut microbiota with COVID-19 vaccination are summarized in **Supplementary Table 1**. Of note, most of them measured antibody response within a relatively short time frame from vaccination, ranging from one to three months (15–18). Several studies also only had small sample size (17–19), while some focused in special patient populations, such as one conducted in infliximab-treated IBD patients (17), and another in people living with HIV (18). In addition, factors that might affect the microbiota, such as diet, were often not statistically accounted for as in our study (15–20). Moreover, some studies employed 16S rRNA gene sequencing instead of shotgun metagenomic sequencing, which had lower resolution and could not profile gut microbiota down to species and strain level (17–19). As a result, these studies found various different bacterial markers that were associated with high vaccine response. Among immunocompetent individuals, these included *Eubacterium rectale* and *Roseburia faecis* in BNT162b2 vaccinees (15), *Bifidobacterium adolescentis* in CoronaVac (inactivated virus vaccine) vaccinees (15), as well as *Collinsella aerofaciens* and *Fusicatenibacter saccharivorans* in BBIBP-CorV (inactivated virus vaccine) vaccinees (16). Different bacterial markers were observed in immunosuppressed individuals. In one study on IBD patients receiving infliximab for >12 weeks, *Bilophila* was found to be enriched in high-responders while *Streptococcus* was enriched in low responders after two doses of BNT162b2 or ChAdOx1 vaccination (17). Another study on people living with HIV found that *Flavonifractor*, *Lachnospira* and *Oscillibacter* were enriched in high-responders after two doses of BNT162b2, while *Butyricimonas* and *Paraprevotella* were enriched in low-responders (18).

Several limitations of our study should be noted. First, our study findings, including the metabolic pathways, were correlative, and further studies on animal models, such as in germ-free mice or in microbiome-modulated animal models (such as through supplementation with a bacteria strain), were required to prove causality behind the association between the gut microbiota, metabolites and vaccine immunogenicity observed in our study. Second, our study findings may not be generalizable to other populations as variation of gut microbiota composition exist across different populations and geographical regions due to various factors, including diet, lifestyle and socioeconomic status (7). Furthermore, the use of extreme case-control design may also have limited the generalizability of our metabolomic findings to other populations. Third, longer follow-up on the durability of immunogenicity after two vaccine doses was not possible as the majority of participants received booster dose after six months.

Nevertheless, we are planning to further investigate whether gut microbiota can predict vaccine immunogenicity and durability after booster doses. The markers identified in this study might potentially apply to immunogenicity after booster doses as well and explain difference in the speed of waning of antibody levels. Moreover, the findings of our study might be translated to clinical applications by developing probiotics and amino acid supplements, which would be validated in animal models and in clinical RCTs on human subjects, to boost vaccine immunogenicity in the general population. In fact, similar studies were done in the past. For instance, one RCT investigated the effect of probiotic supplementation with *Limosilactobacillus reuteri* DSM 17938 for 6 months on serum antibody levels after COVID-19 vaccination, and found that probiotic supplementation exhibited significantly higher serum antibody levels than placebo arm at >28 days after vaccination (84). Thus, we were hopeful that our study could help to lay the foundation for further animal studies or even RCTs to validate and investigate the potential gut bacterial species that were implicated in enhancing durable vaccine immunogenicity for potential therapeutic use in the future.

## Conclusion

Certain gut bacterial species could be associated with persistently high vaccine immunogenicity at six months after two doses of BNT162b2. These results may potentially facilitate the development of gut microbiota interventions to improve long-term durability of vaccine immune response.

## Data Availability

All sequencing data generated in this study are deposited in the Sequence Read Archive under BioProject PRJNA983517.
