# Supplementary material for: Gut microbiota is associated with persistence of longer-term BNT162b2 vaccine immunogenicity
Source: Front Immunol. 2025 Feb 27;16:1534787. doi: 10.3389/fimmu.2025.1534787 (PMC11903479; doi:10.3389/fimmu.2025.1534787)
Supplement: Supplementary file 1 [file DataSheet1.docx]

**SUPPLEMENTARY METHODS**

***Targeted quantitation of short-chain fatty acids (SCFAs)***

Sample processing and gas chromatography-tandem mass spectrometry (GC-MS/MS) analysis were performed at Centre for PanorOmic Sciences - Proteomics and Metabolomics Core, LKS Faculty of Medicine, University of Hong Kong.

*Materials*

Acetic acid (C2:0), propionic acid (C3:0), butyric acid (C4:0), isobutyric acid (C4:0i), valeric acid (C5:0), isovaleric acid (C5:0i), hexanoic acid (C6:0), 4-methylvaleric acid (C6:0i), heptanoic acid (C7:0) and the internal standards 3-methylvaleric acid were purchased from Sigma-Aldrich. Diethyl ether was purchased from VWR. Water was purchased from Wako. Concentrated hydrochloric acid (HCl) was purchased from Fisher Chemical. Methyl tert-Butyl Ether (MTBE) was purchased from Labscan. All reagents were in HPLC grade equivalent or higher. Ultra high purity (>99.999%) nitrogen and helium gas were purchased from Linde HKO Ltd.

*Sample preparation*

Extraction

8 µl of 0.5 M HCl with 20 nmol 3-methylvaleric acid internal standard were added in 20 µl plasma sample. 50 µL of MTBE was added to the sample extract and vortexed for 10 sec to emulsify. After phase separation, 1 µl of upper layer MTBE was injected for GC-MS/MS analysis.

*Data acquisition*

GC-MS/MS chromatogram was acquired in SCAN and SIM mode in an Agilent 7890B GC - Agilent 7010 Triple Quadrapole Mass Spectrometer system. The sample was separated through a Phenomenex Zebron ZB-WAX capillary column (30 m × 0.25 mm ID, 0.25 µm film thickness) under constant flow of helium at 1 mL min−1. The GC oven program started at 100°C (hold time 2 min) and was increased to 210°C at a ramp rate of 20°C/min (hold time 2 min). Characteristic fragment ions (m/z 43, 45, 60, 73, 74, 87, 88) were monitored in SIM mode throughout the run. Mass spectra from m/z 40-350 were acquired in SCAN mode.

*Data analysis*

Data analysis was performed using the Agilent MassHunter Workstation Quantitative Analysis Software. Linear calibration curves for each analyte were generated by plotting peak area ratio of external/internal standard against standard concentration at different concentration levels. Analytes were confirmed by comparing the retention time and ratio of characteristic fragment ions between the sample and standard.

***Targeted quantitation of isoleucine***

Liquid chromatography-tandem mass spectrometry (LC-MS/MS) analysis was performed at Centre for PanorOmic Sciences - Proteomics and Metabolomics Core, LKS Faculty of Medicine, University of Hong Kong.

*Materials*

Water and methanol were purchased from Wako. Acetonitrile was purchased from Honeywell. Isoleucine, ammonium formate and internal standard chlorophenylalanine were purchased from Sigma-Aldrich. Formic acid was purchased from Fisher Chemical. All reagents were in HPLC grade equivalent or higher.

*Sample preparation*

For fluid sample, 1 ml ice-cold 3:3:2 acetonitrile:isopropanol:water was added to the 30 µl of sample. The sample was vortexed for 10 s, shaked for 5 min at 4℃ and then centrifuged for 2 min at 14,000 rcf. 333 μl of supernatant was dried under a gentle stream of nitrogen at room temperature.

The sample was then reconstituted with 50 µl 8:2 acetonitrile:water with 100 ppb chlorophenylalanine internal standard. Then, 5 μL was injected into the LC-MS/MS system.

*Data acquisition*

LC-MS/MS

The chromatographic separation was carried out on an Vanquish UPLC (Thermo Fisher, Waltham, MA, USA). Mobile phases used were 10mM ammonium formate with 0.1% formic acid in acetonitrile and water, v/v 6:4 (A) and 9:1 (B). The column was a Waters BEH amide (2.1×100 mm, 1.7 μm). An injection volume of 5 μL and a flow rate of 0.35 mL min−1 were used. The column oven temperature was set at 40°C. The gradient started at 100% B and was held for 0.5 min decreased linearly to 0% over 13 min, and held for 1.5 min, increased linearly to 100% B for re-equilibration time at starting conditions.

The mass spectrometry analysis was processed using an Orbitrap Exploris 120 mass spectrometer Thermo Fisher (Waltham, MA, USA) equipped with a HESI II probe in polar switching mode with source parameters set as follows: sheath gas flow rate, 60; auxiliary gas flow rate, 17; sweep gas flow rate, 1; spray voltage, +3.5/-3.0 kV; capillary temperature, 275 oC; S-lens RF level, 70; and heater temperature, 325 °C. Data was collected at dd-MS2 mode.

*Data analysis*

Data analysis was performed using the Xcalibur and Freestyle TraceFinder (Thermofisher Scientific, Waltham, USA) software. Linear calibration curves for each analyte were generated by plotting peak area ratio of external/internal standard against standard concentration at different concentration levels. Analytes were confirmed by comparing the retention time, accurate mass and characteristic fragment mass between the sample and standard.

***Machine learning model***

This was a binary classification problem using random forest algorithm, which is made of an ensemble of decision trees and has the advantages of being robust to noise and being able to capture nonlinear interactions. The objective was to predict the vaccine response status (high-response vs low response) at 180 days after the administration of two doses of vaccination using the variables with p<0.15 in either univariate logistic regression or univariate linear regression. The predicted outcomes were labelled as “0” (low response) or “1” (high response).

Hyperparameter tuning was performed using grid search (3^p^ grid size; p represents the number of hyperparameters) with 3-fold cross-validation to optimize key parameter (e.g. mtry). This involved partitioning the training data into three equally-sized subsets, training the model on two of the subsets and validating it on the remaining subset, and then repeating this process 5 times with validation subset. Area under receiver operating characteristic curve (AUC) on the validation set was then obtained.

**Supplementary Table 1: Published clinical studies investigating association between gut microbiota and COVID-19 vaccine immunogenicity**

| **Study** | **Region** | **Sample size/Vaccine type & doses** | **Study population** | **Timepoint for gut microbiota profiling** | **Timepoint for antibody measurement** | **Sequencing method** | **Method for measuring antibody level** | **Statistical adjustment for diet and/or antibiotics** | **Major findings of gut microbiota** | |
| --- | --- | --- | --- | --- | --- | --- | --- | --- | --- | --- |
|  |  |  |  |  |  |  |  |  | **Enriched in high-responder** | **Enriched in low-responder** |
| Ng et al, 2022^1^ | Hong Kong, China | BNT162b2 (mRNA vaccine): n=101; 2 doses  CoronaVac  (inactivated virus vaccine): n=37; 2 doses | Mainly immune-competent adults (3% of BNT162b2 cohort received immune-modulator) | At baseline before vaccination | At one month after 2^nd^ dose of vaccine | Shotgun metagenomic sequencing | SARS-CoV-2 surrogate virus neutralisation test (sVNT) | No | BNT162b2:   - *Eubacterium rectale* - *Roseburia faecis* - *Bacteroides thetaiotaomicron* - *Bacteroides sp OM05-12*   CoronaVac:   - *Bifidobacterium adolescentis* | CoronaVac:   - *Bacteroides vulgatus* - *Bacteroides thetaiotaomicron* - *Ruminococcus gnavus* |
| Tang et al, 2022^2^ | China | BBIBP-CorV (inactivated virus vaccine): n=207; 2 doses | Immuno-competent adults | At baseline before vaccination | At day 14 after second dose (day 42 from first dose) | Shotgun metagenomic sequencing | Magnetic chemiluminescence enzyme immunoassay (MCLIA) kits | No | - *Collinsella aerofaciens* - *Fusicatenibacter saccharivorans* - *Eubacterium ramulus* - *Veillonella dispar* | - *Lawsonibacter asaccharolyticus* |
| Alexander et al, 2023^3^ | United Kingdom | BNT162b2 (mRNA vaccine): n=28; 2 doses  ChAdOx1 (adenoviral-vector-based vaccine): n=15; 2 doses | Inflammatory bowel disease (IBD) patients receiving infliximab for > 12 weeks | At baseline before vaccination | Between 14 and 100 days after second dose of vaccination  (subjects had serological analyses at 8-week intervals, and study endpoint was taken as first time-point measured post-2^nd^ dose, but no earlier than 14 days after 2^nd^ dose) | 16S rRNA gene sequencing | Roche Elecsys Anti-SARS-CoV-2 spike (S) electro-chemiluminescence immunoassay | No | - *Bilophila* | - *Streptococcus* |
| Ray et al, 2023^4^ | Sweden | BNT162b2 (mRNA vaccine): n=75; 2 doses | Immuno-competent adults | At baseline before vaccination | Day 35 after first dose vaccine | 16S rRNA gene sequencing | Roche Elecsys Anti-SARS-CoV-2 spike (S) electro-chemiluminescence immunoassay | No | - *Bacteroides* - *Sutterella* - *Lachnospiraceae FCS020 group* | - *Alloprevotella* - *Anaerofilum* - *Succinivibrio* - *Moryella* - *Negativibacillus* - Certain members of the family *Ruminococcaceae* |
|  |  | BNT162b2 (mRNA vaccine): n=68; 2 doses | People living with HIV (PLWH) | At baseline before vaccination | Day 35 after first dose vaccine | 16S rRNA gene sequencing | Roche Elecsys Anti-SARS-CoV-2 spike (S) electro-chemiluminescence immunoassay | No | - *Flavonifractor* - *Lachnospira* - *Oscillibacter* | - *Butyricimonas* - *Paraprevotella* |
| Peng et al, 2023^5^ | Hong Kong, China | BNT162b2 (mRNA vaccine): n=121; 2 doses  CoronaVac (inactivated virus vaccine): n=40; 2 doses | Immuno-competent adults | At baseline before vaccination | At 6 months after 2^nd^ dose vaccine | Shotgun metagenomic sequencing | SARS-CoV-2 surrogate virus neutralisation test (sVNT) | No | BNT162b2:   - - *Roseburia faceis*   - *Bifidobacterium adolescentis*   - *Bifidobacterium bifidum*   CoronaVac:   - - *Phocaeicola dorei*   - *Blautia massiliensis*   - *Dorea formicigenerans* | CoronaVac:   - *F. praustnitzii* |
| Seong et al, 2024^6^ | Korea | BNT162b2 (mRNA vaccine): n=23; 3 doses  ChAdOx1 (adenoviral-vector-based vaccine): n=21; 2 doses | Immuno-competent adults | 3 weeks after 2^nd^ dose | Antibody level measured at:  - 3 weeks after 2^nd^ dose  - 6 months after 2^nd^ dose and before booster dose (for BNT162b2) | 16S rRNA gene sequencing | Roche Elecsys Anti-SARS-CoV-2 spike (S) electro-chemiluminescence immunoassay | No | ***Enriched in slow-decay group***  BNT162b2:   - *Faecalibacterium praustnitzii*   ChAdOx1:   - *Escherichia coli* | |

**Supplementary Table 2. Univariate linear regression between clinical factors and NAb level**

|  |  |  | **Univariate linear regression** | | |
| --- | --- | --- | --- | --- | --- |
|  |  |  | **Beta** | **95% CI** | **p-value** |
| Age ≥ 55 years |  |  | -9.5 | -42, 23 | 0.568 |
| Male sex |  |  | -18 | -49, 13 | 0.251 |
| Adequate level of exercises^†^ |  |  | 21 | -9.1, 51 | 0.169 |
| Diet (rPDQS score at 3^rd^ tertile) |  |  | -0.99 | -32, 30 | 0.949 |
| Smoking |  |  | -48 | -116, 19 | 0.159 |
| Overweight or obese |  |  | -29 | -58, 0.79 | 0.056 |
| Diabetes mellitus |  |  | -54 | -113, 4.2 | 0.069 |
| Triglycerides |  |  | 4.2 | -21, 30 | 0.746 |
| Total cholesterol |  |  | 4.6 | -11, 20 | 0.559 |
| Low-density lipoprotein |  |  | 5.5 | -13, 24 | 0.560 |
| Hepatic steatosis |  |  | 7.5 | -23, 38 | 0.625 |
| Proton pump inhibitor use |  |  | -21 | -68.3, 26.3 | 0.383 |
| Antibiotic use |  |  | -6.2 | -58.3, 46 | 0.816 |

^†^adequate level of exercises is defined as meeting WHO recommendation (at least 150-300 minutes of moderate-intensity, or 75-150 minutes of vigorous-intensity aerobic exercise per week)

Abbreviations: 95% CI, 95% confidence interval; OWOB, overweight or obese; WHO, World Health Organization; rPDQS, rapid prime diet quality score; NAb, neutralizing antibody

**Supplementary Table 3. Sensitivity analysis of association between high vaccine response and a combination of clinical factors (incorporating overweight or obese) and bacterial species using multivariate logistic regression**

|  | **aOR** | **95% CI** | **p-value** |  |
| --- | --- | --- | --- | --- |
| Age ≥ 55 years | 0.64 | 0.29, 1.31 | 0.232 |  |
| Overweight or obese | 1.02 | 0.55, 1.89 | 0.956 |  |
| Diabetes mellitus | 0.21 | 0.01, 1.12 | 0.139 |  |
| *Ruminococcus bicirculans****** | 1.85 | 1.01, 3.47 | 0.050 |  |
| *Parasutterella excrementihominis****** | 2.24 | 1.21, 4.26 | 0.012 |  |
| *Streptococcus salivarius****** | 2.03 | 1.10, 3.84 | 0.025 |  |
|  |  |  |  |  |

***** High relative abundance is defined as relative abundance higher than median of the cohort

Abbreviations: aOR, adjusted odds ratio; 95% CI, 95% confidence interval

**Supplementary Table 4: p-trend analysis of NAb level at day 180 stratified using baseline relative abundance of *Parasutterella excrementihominis* and DM status**

|  | **NAb level at day 180*** | **p-trend** |
| --- | --- | --- |
|  |  | 0.022 |
| High-NDM | 81.7 (37.8, 165.0) |  |
| High-DM | 50.3 (28.2, 64.6) |  |
| Low-NDM | 45.1 (27.6, 91,6) |  |
| Low-DM | 27.8 (19.9, 37.0) |  |
|  |  |  |

*NAb level at day 180 expressed as median (IQR)

Note: High abundance is defined as higher than or equal to the median relative abundance of the species

Abbreviations: NAb, neutralizing antibody level to SARS-CoV-2; DM, diabetes mellitus; NDM, non-diabetes mellitus

**Supplementary Table 5: p-trend analysis of NAb level at day 180 stratified using baseline relative abundance of *Parasutterella excrementihominis* and OWOB status**

|  | **NAb level at day 180*** | **p-trend** |
| --- | --- | --- |
|  |  | 0.009 |
| High-NW | 101.0 (46.5, 165.0) |  |
| High-OWOB | 68.0 (36.4, 139.0) |  |
| Low-NW | 42.7 (26.8, 91.6) |  |
| Low-OWOB | 45.2 (25.5, 83.7) |  |
|  |  |  |

*NAb level at day 180 expressed as median (IQR)

Note: High abundance is defined as higher than or equal to the median relative abundance of the species

Abbreviations: NAb, neutralizing antibody level to SARS-CoV-2; OWOB, overweight or obese**;** NW, normal weight

**Supplementary Table 6. Subgroup analysis of association between high vaccine response and a combination of clinical factors (incorporating overweight or obese) and bacterial species using multivariate logistic regression based on sex**

|  | **Male** | | |  |  | **Female** |  |
| --- | --- | --- | --- | --- | --- | --- | --- |
|  | **aOR** | **95% CI** | **p-value** |  | **aOR** | **95% CI** | **p-value** |
| Age ≥ 55 | 0.20 | 0.03, 0.86 | 0.054 |  | 0.95 | 0.38, 2.27 | 0.914 |
| Diabetes mellitus | 0.49 | 0.02, 3.88 | 0.552 |  | 0.00 | NA | 0.991 |
| *Ruminococcus bicirculans* | 1.52 | 0.51, 4.58 | 0.451 |  | 2.36 | 1.09, 5.35 | 0.034 |
| *Parasutterella excrementihominis* | 4.59 | 1.51, 15.7 | 0.01 |  | 1.55 | 0.71, 3.41 | 0.272 |
| *Streptococcus salivarius* | 1.59 | 0.53, 5.10 | 0.419 |  | 2.29 | 1.07, 5.03 | 0.035 |

***** High relative abundance is defined as relative abundance higher than median of the cohort

Abbreviations: aOR, adjusted odds ratio; 95% CI, 95% confidence interval

**Supplementary Table 7. Summary of metabolic pathways and their linear discriminant analysis (LDA) scores**

| **Abbreviation** | **Full name** | **Vaccinee group** | **LDA score** |
| --- | --- | --- | --- |
| **Biosynthesis** | | | |
| THISYNARA-PWY | Superpathway of thiamine diphosphate biosynthesis III (eukaryotes) | High responders | 2.36349963 |
| PWY-6292 | Superpathway of L−cysteine biosynthesis (mammalian) | High responders | 2.251804585 |
| PWY-3001 | Superpathway of L−isoleucine biosynthesis I | High responders | 2.158237939 |
| PWY-6168 | Flavin biosynthesis III (fungi) | Low responders | -2.279090042 |
| **Degradation/Utilization/Assimilation** | | | |
| PWY-621 | Sucrose degradation III (sucrose invertase) | High responders | 2.331307495 |
| PWY-5384 | Sucrose degradation IV (sucrose phosphorylase) | High responders | 2.296973352 |
| PWY-6317 | D−galactose degradation I (Leloir pathway) | High responders | 2.291606427 |
| PWY-5136 | Fatty acid & β−oxidation II (plant peroxisome) | Low responders | -2.005459655 |
| P441-PWY | Superpathway of N−acetylneuraminate degradation | Low responders | -2.113981067 |
| P461-PWY | Hexitol fermentation to lactate, formate, ethanol and acetate | Low responders | -2.126896119 |
| PWY0-1477 | Ethanolamine utilization | Low responders | -2.168246915 |

**Supplementary Figure 1. Comparison of different baseline alpha diversity indexes of gut microbiota between high- and low-vaccine responders**


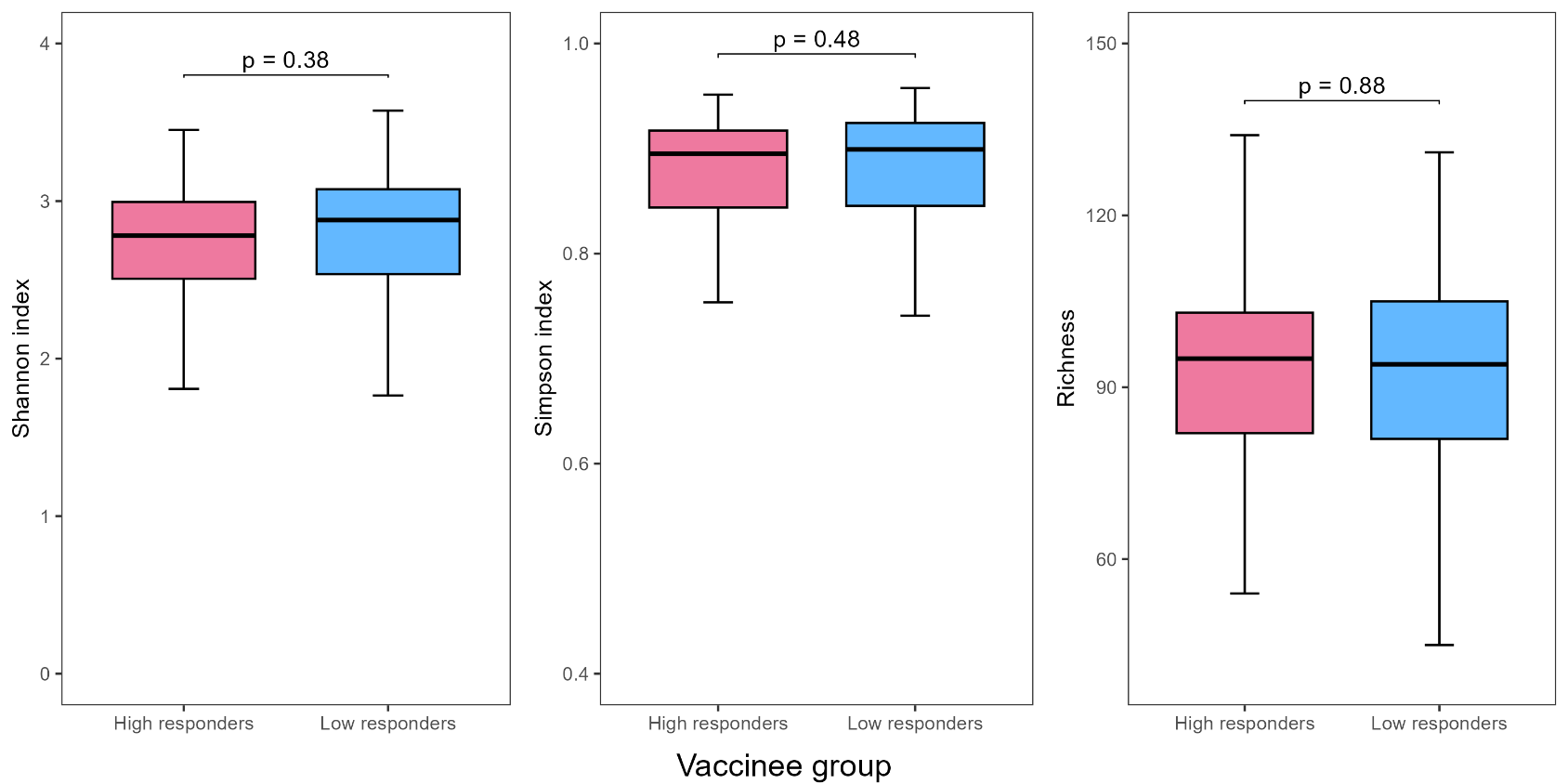


**Supplementary Figure 2. Comparison of baseline beta diversity between high and low responders** **between high- and low-vaccine responders**


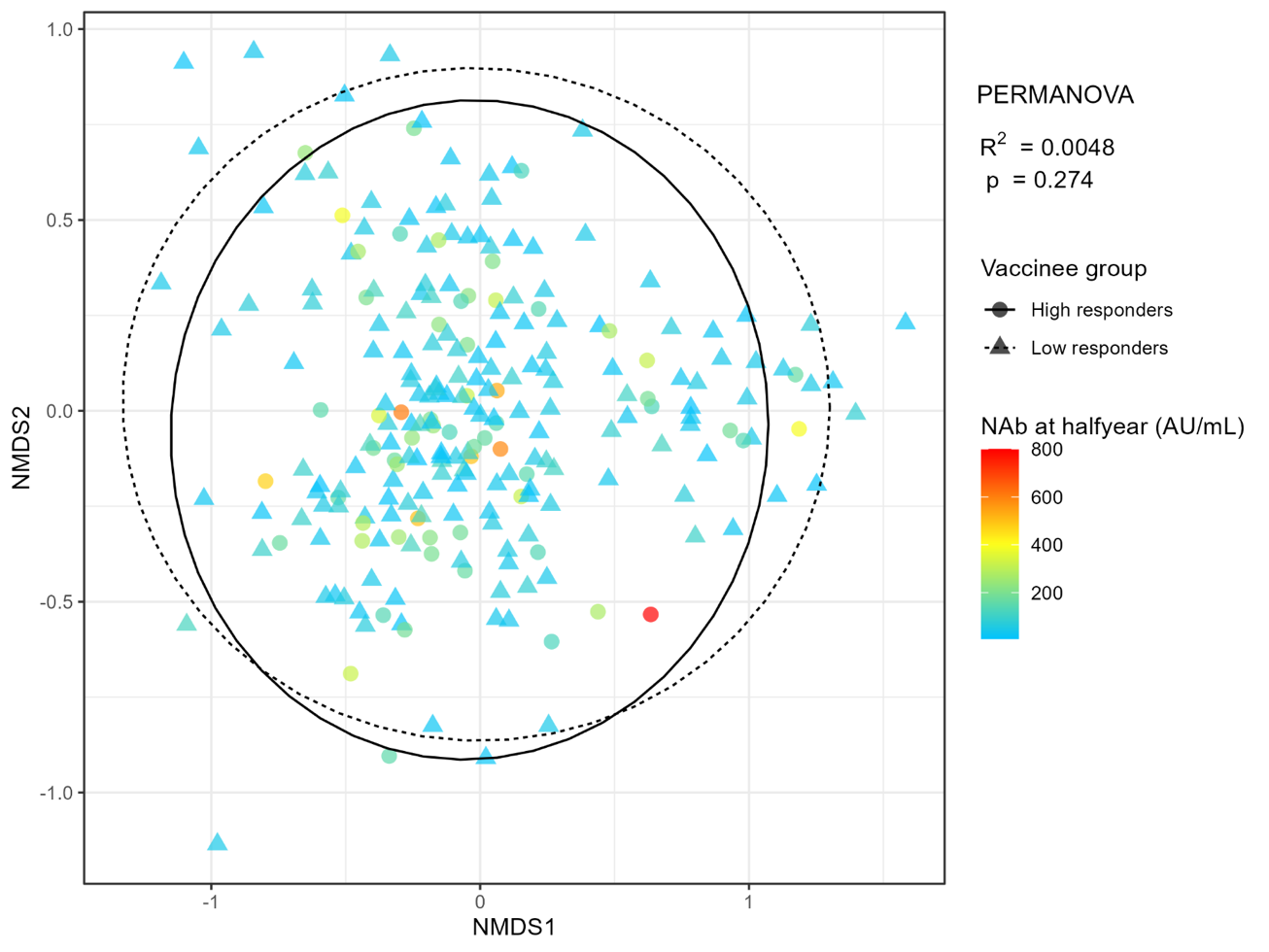


**Supplementary Figure 3: AUC of clinical factors (age ≥ 55 years, DM and OWOB) and the and bacterial species factors (*R. bicirculans, P. excrementihominis, S. salivarius*) in predicting vaccine response using random forest supervised machine learning model**

***
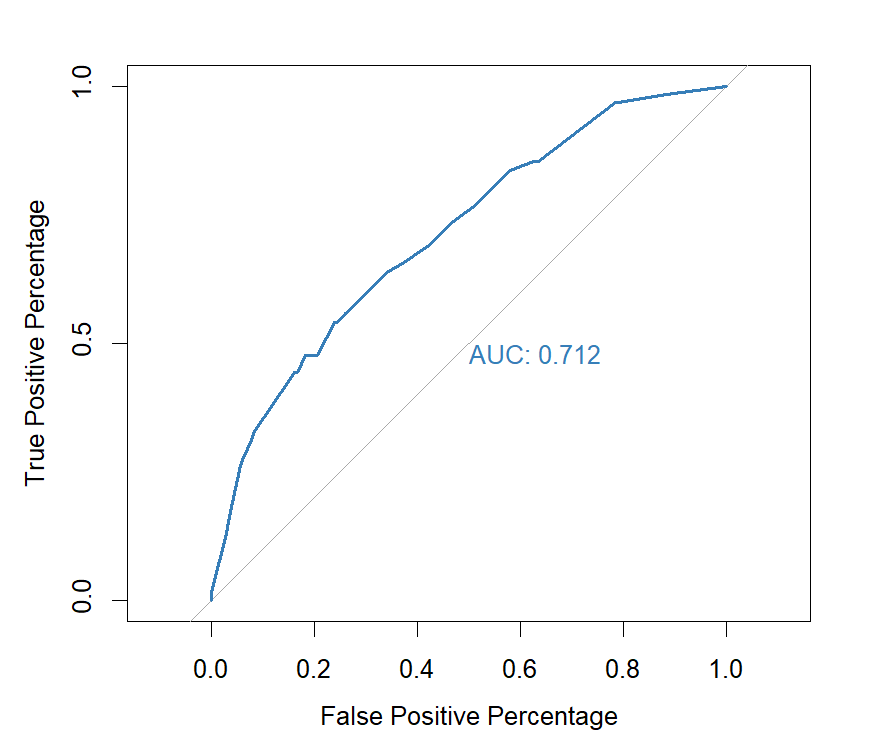
***

Abbreviations: AUC, area under receiver operating characteristic curve

**Supplementary Figure 4. Relative abundances of baseline gut bacterial species according to diabetes mellitus and overweight-or-obese status**

**
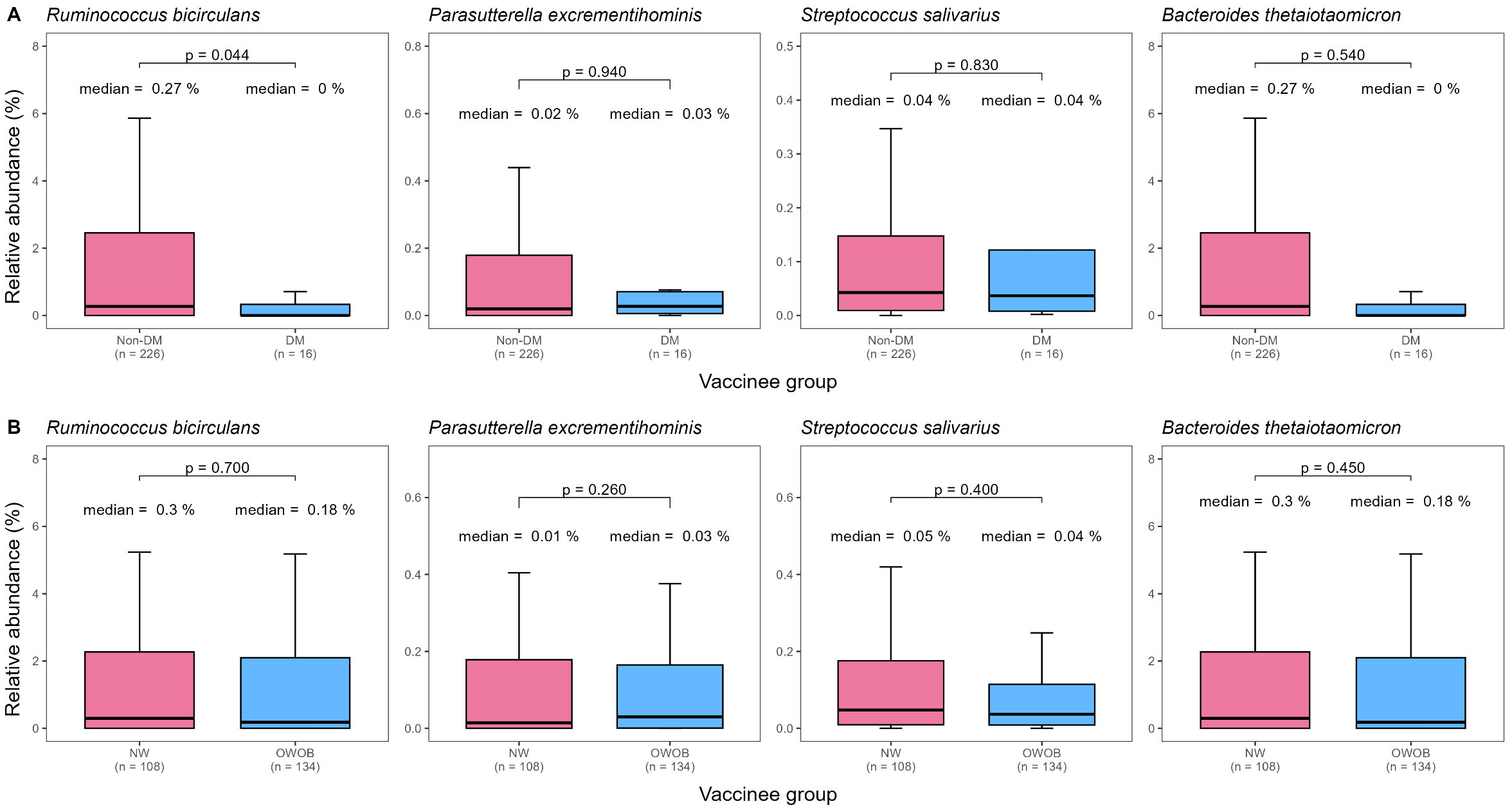
**

Abbreviations: DM, diabetes mellitus; NDM, non-diabetes mellitus; NW, normal weight; OWOB, overweight or obese

**Supplementary Figure 5. NAb level at day 180 stratified using bacterial markers’ baseline relative abundance and DM status**


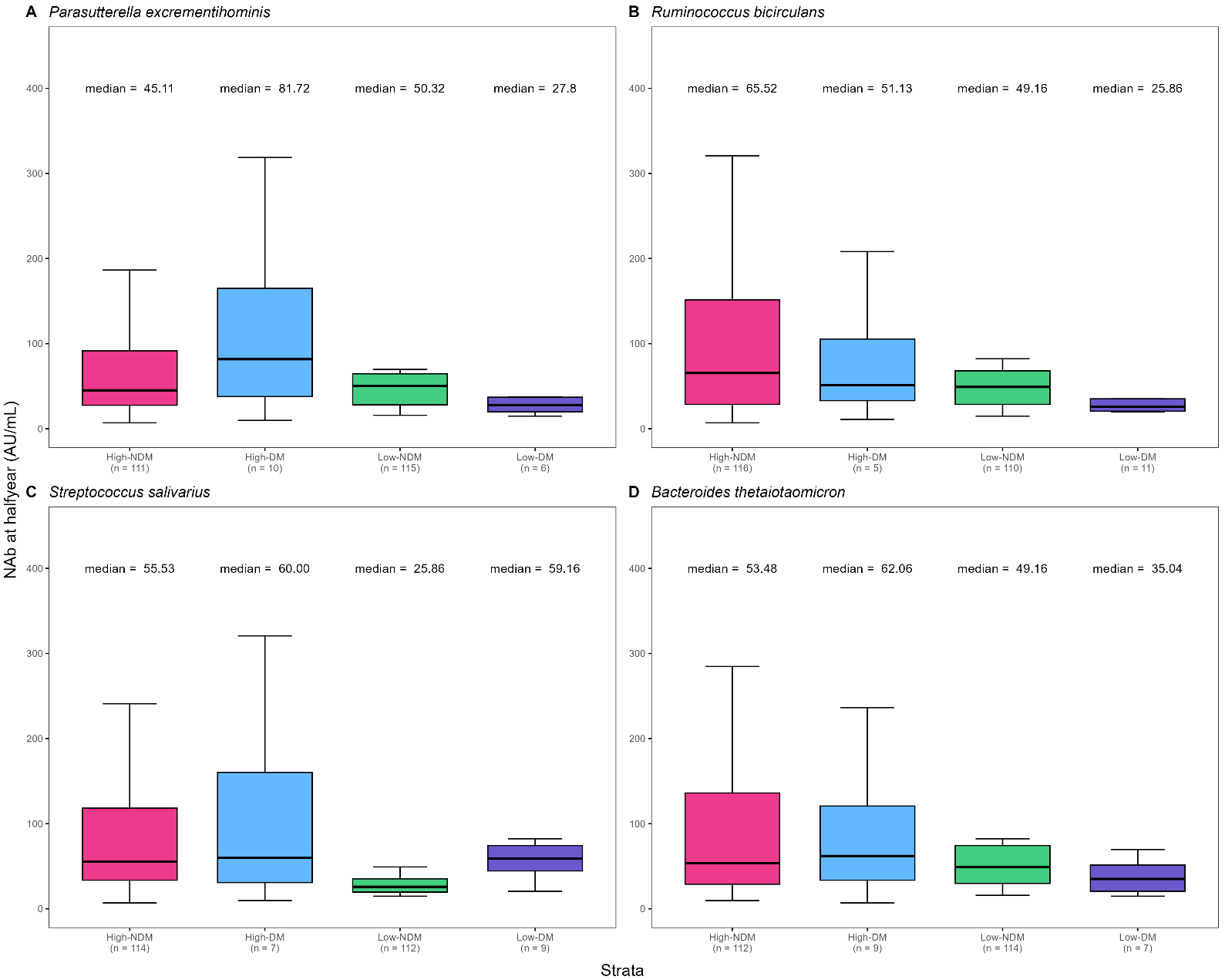


Note: High abundance is defined as higher than or equal to the median relative abundance of the species

Abbreviations: DM, diabetes mellitus; NDM, non-diabetes mellitus; NAb, neutralizing antibody level to SARS-CoV-2

**Supplementary Figure 6. NAb level at day 180 stratified using bacterial markers’ baseline relative abundance and overweight-or-obese status**


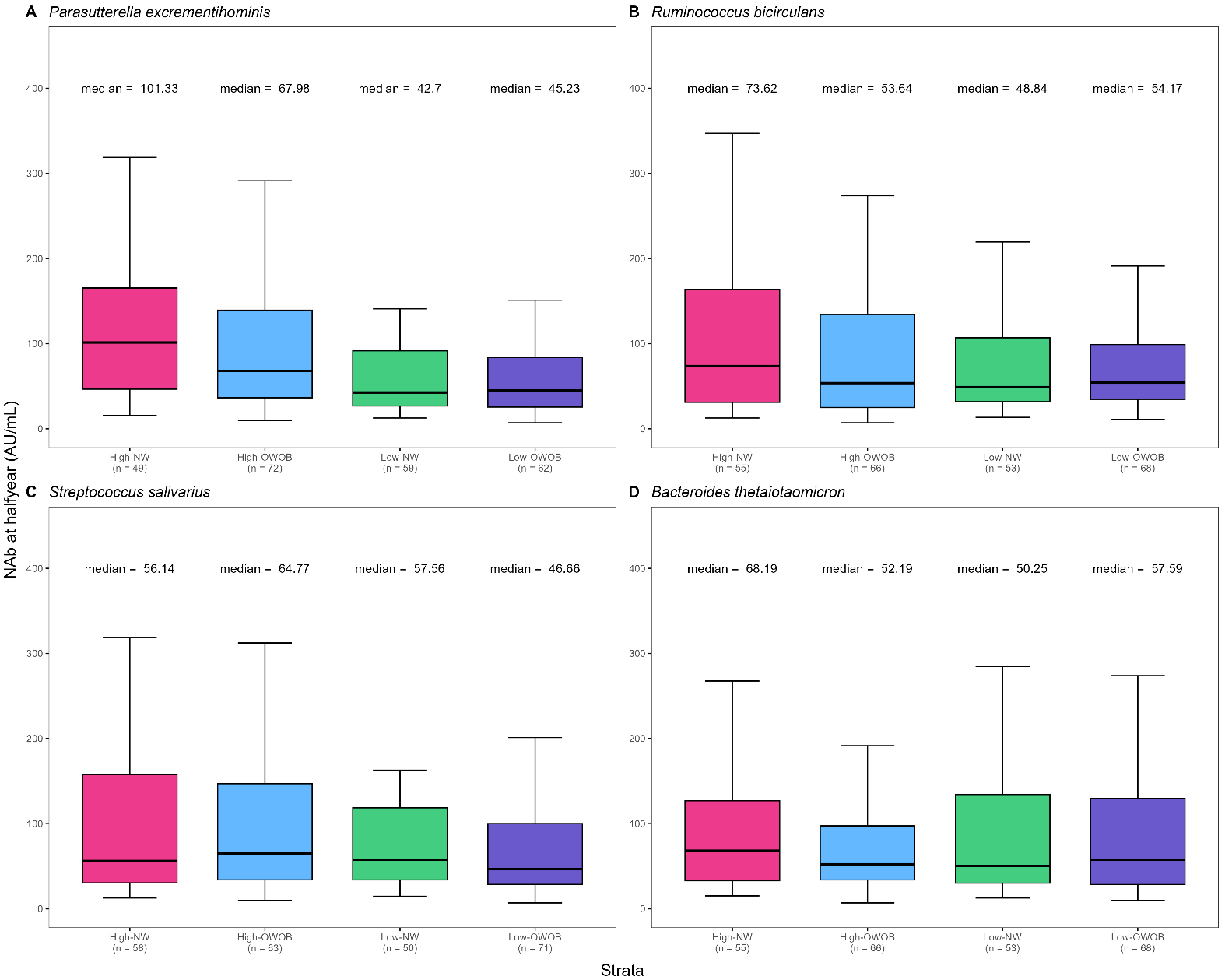


Note: High abundance is defined as higher than or equal to the median relative abundance of the species

Abbreviations: NW, normal weight; OWOB, overweight or obese; NAb, neutralizing antibody level to SARS-CoV-2

**Supplementary Figure 7. Day 180 NAb level stratified using baseline *Parasutterella excrementihominis* relative abundance, DM and overweight-or-obese status**


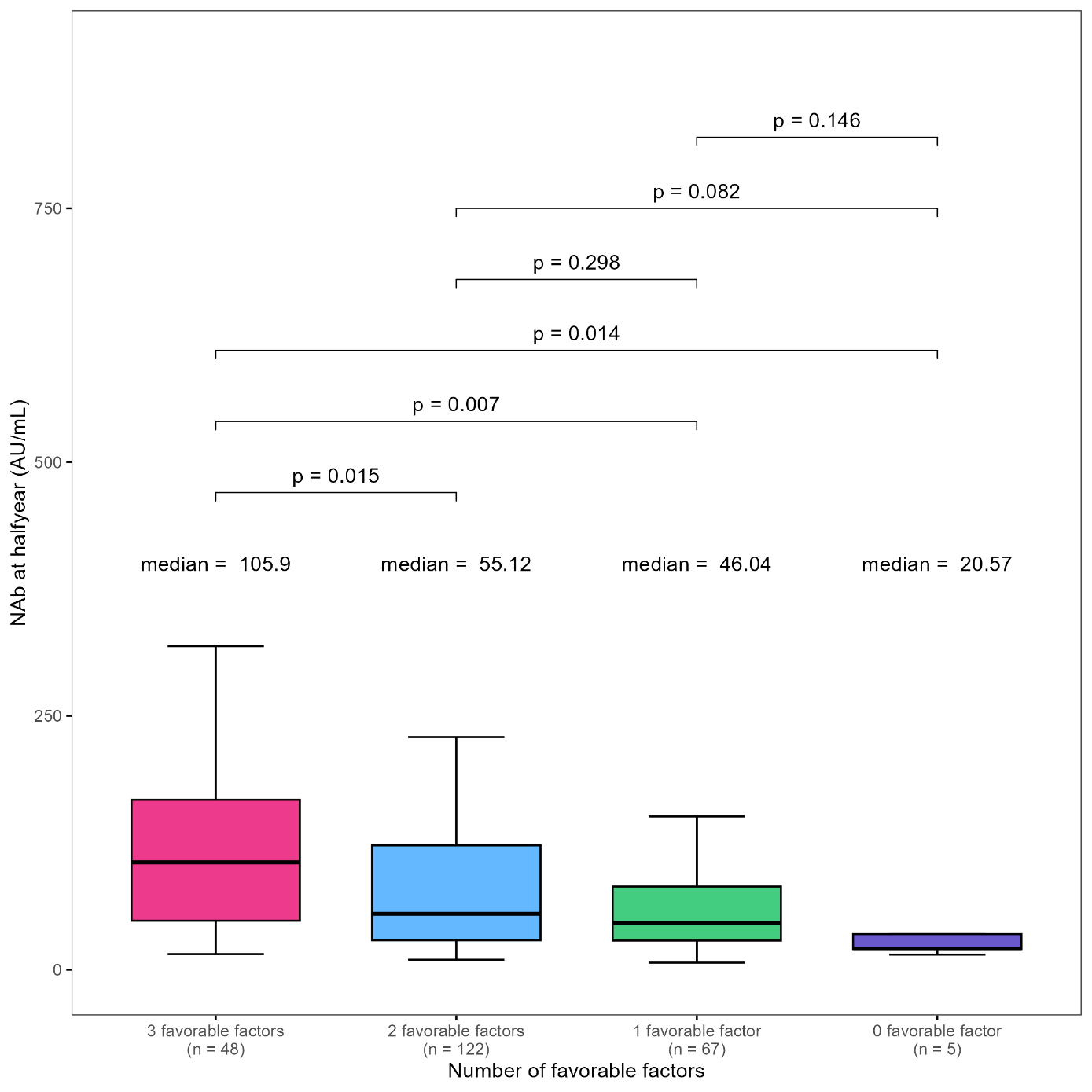


Note: Favorable factors are high relative abundance of baseline gut *Parasutterella excrementihominis,* absence of diabetes mellitus and absence of overweight and obese (OWOB)

**Supplementary Figure 8. Baseline metabolic pathways enriched in high-responders vs low responders of BNT162b2 vaccine at day 180**


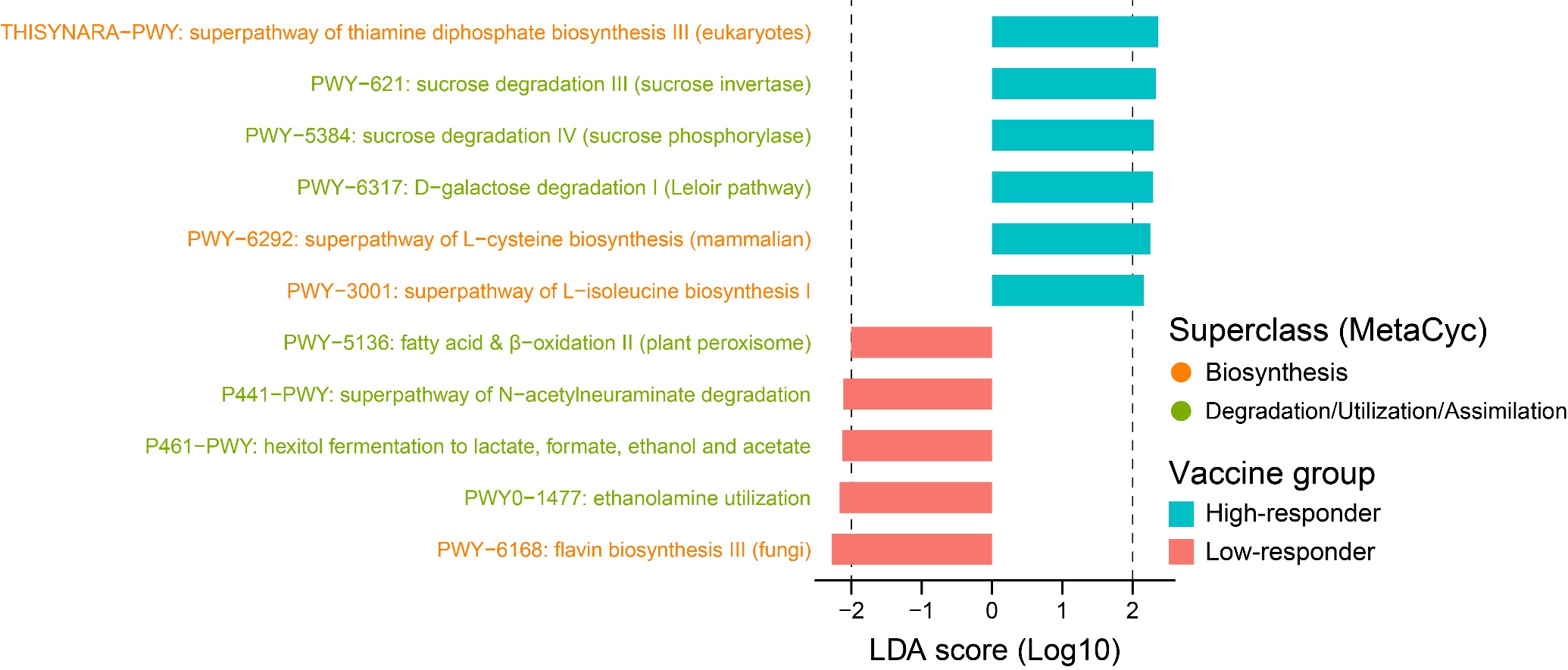


References

1. Ng SC, Peng Y, Zhang L, et al. Gut microbiota composition is associated with SARS-CoV-2 vaccine immunogenicity and adverse events. Gut 2022;71:1106-1116.

2. Tang B, Tang L, He W, et al. Correlation of gut microbiota and metabolic functions with the antibody response to the BBIBP-CorV vaccine. Cell Rep Med 2022;3:100752.

3. Alexander JL, Mullish BH, Danckert NP, et al. The gut microbiota and metabolome are associated with diminished COVID-19 vaccine-induced antibody responses in immunosuppressed inflammatory bowel disease patients. EBioMedicine 2023;88:104430.

4. Ray S, Narayanan A, Vesterbacka J, et al. Impact of the gut microbiome on immunological responses to COVID-19 vaccination in healthy controls and people living with HIV. NPJ Biofilms Microbiomes 2023;9:104.

5. Peng Y, Zhang L, Mok CKP, et al. Baseline gut microbiota and metabolome predict durable immunogenicity to SARS-CoV-2 vaccines. Signal Transduct Target Ther 2023;8:373.

6. Seong H, Yoon JG, Nham E, et al. The gut microbiota modifies antibody durability and booster responses after SARS-CoV-2 vaccination. J Transl Med 2024;22:827.
